# Case Series on DRESS: An Unpredictable Adverse Drug Reaction

**DOI:** 10.31138/mjr.34.2.245

**Published:** 2023-06-30

**Authors:** Maryam Fatima, Salwa Sahar Azimi, Soumya Ashwini, Madhuri H Radhakrishna

**Affiliations:** 1 AIG Hospitals,; 2 G. Pulla Reddy College of Pharmacy

**Keywords:** maculopapular rash, lymphadenopathy, vulnerability, offending drug, corticosteroids

## Abstract

Drug rash with eosinophilia and systemic symptoms syndrome (DRESS) is a potentially life-threatening, drug-induced, multi-organ system reaction. The most frequently involved organ is the liver, followed by the kidneys and lungs. Early detection and diagnosis followed by withdrawal of the offending agent is vital to minimise the associated morbidity and mortality, and a detailed drug history is vital to identify the causative drugs. Although Spanish guidelines were developed by a panel of allergy specialists from the Drug Allergy Committee of the Spanish Society of Allergy and Clinical Immunology (SEAIC) and are available in literature from 2020, many clinicians are still unaware about the management of this syndrome. Framing National guidelines for the early diagnosis and pharmaco-therapeutic management of DRESS will help healthcare professionals to save the patients from unintended vulnerability. We hereby present a case series on DRESS.

## INTRODUCTION

DRESS (Drug Rash with Eosinophilia and Systemic Symptoms) is a severe drug-induced hypersensitivity syndrome characterised by diffuse maculopapular rash, lymphadenopathy, multivisceral involvement, eosinophilia, and atypical lymphocytes with a mortality rate of 10–40%. DRESS is an uncommon severe adverse drug reaction.^1^ Although it is important to recognise the syndrome as early as possible to save the patients from unintended vulnerability, many clinicians fail to identify DRESS due to delayed onset of symptoms,^2^ involvement of one or multiple organs, and the clinical features overlapping with other severe cutaneous adverse reactions (SCARs).^3^ Early diagnosis and prompt treatment following identification and withdrawal of offending drugs is necessary to provide better pharmaceutical care decreasing the risk of morbidity and mortality.^4^ A detailed medical and medication history is required to minimise complications from the syndrome. For the diagnosis of DRESS two major criteria has been developed by international standard group, ie, RegiSCAR and J-SCAR.^5^ In our study we have considered RegiSCAR criteria to identify DRESS. The mainstay of treatment is the use of systemic corticosteroids, but other options such as intravenous immunoglobulin, cyclosporine, mycophenolate mofetil, rituximab, and cyclophosphamide have been described.^6^ Spanish guidelines have been developed for DRESS, which may prove to be useful for the clinicians to identify DRESS and manage the condition appropriately.^7^

## ETIOLOGY

The most common causative agents include antiepileptics like carbamazepine, lamotrigine, or phenytoin in 35%, NSAIDs in 13%, sulphonamides like Sulfasalazine, Dapsone, Trimethoprim-sulfamethoxazole, or Sulfadiazine in 12%, antibiotics such as Vancomycin, Minocycline, or Penicillin in 11%, and Allopurinol in 6% of cases.^8^ Recently, we found reports on Lamotrigine, Carbamazepine, Vancomycin, Minocycline, Ranitidine, Hydroxychloroquine, Raltegravir, Teicoplanin, Piperacillintazobactam, Ciprofloxacin, Meropenem, Co-trimoxazole, Amoxicillin-clavulanate. Most commonly, medicines associated with DRESS are anticonvulsants, Beta lactam, and Allopurinol. Other medicines that are known to be associated with DRESS are NSAIDS, Captopril, anti RVTs.^9^ The incidence of DRESS with anticonvulsants has been reported in 1 in 5000 to 1 in 10000 exposures.^10^

## CASE REPORT 1

**Figure 1.**
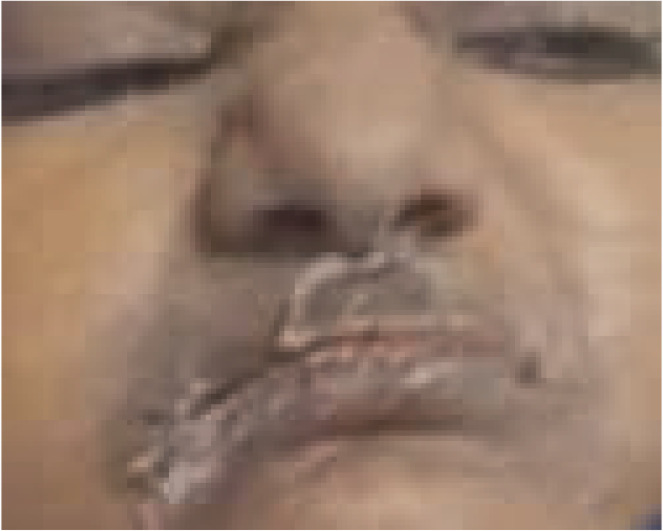
Mucosal involvement of the lesions at the angles of the mouth.

**Figure 2.**
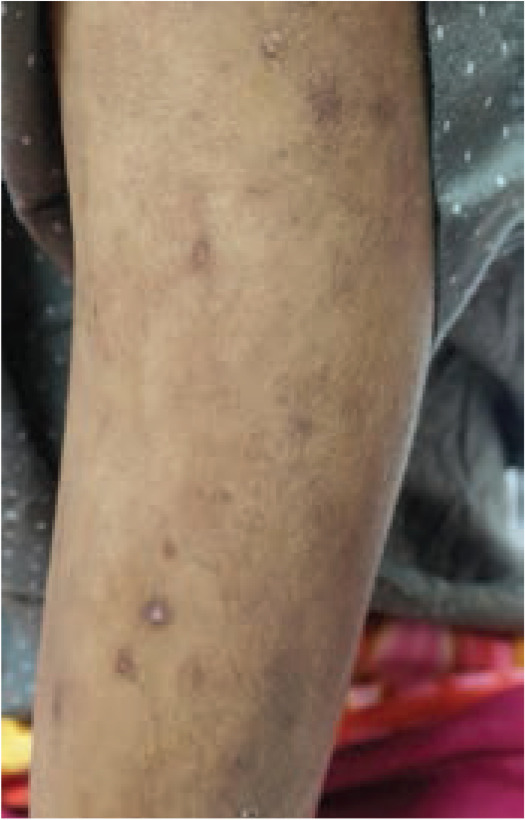
Development of rashes on the arm due to Leflunomide.

A 32-year-old lady presented with complaints of a rash with itching for 20 days, fever for 17 days, vomiting, and loose stools for 1 day. The rash had started from the forearm, legs, and spread to the entire body. It was a maculopapular rash, mucosal involvement at the angles of the mouth. On examination, she was lethargic, febrile, and icterus was present, facial oedema was present with crustings at the angle of mouth and right side of hip. Systemic examination was otherwise normal. The detailed laboratory investigations are given in **Table 1**. Upon taking past medication history, the patient revealed that she had taken ayurvedic medicines for jaundice, but it did not subside. Hence, she consulted a local hospital where she was treated with Doxycycline, Ceftriaxone, Udiliv, Hepkart. She had joint pains for which Aceclofenac, Paracetamol, Rabeprazole, Domperidone, Deflazacort, and Leflunomide was advised for 1 month in suspicion of arthritis. After few weeks of medication intake she developed rashes, recurrent fever spikes with itching, With this detailed drug literature and results of laboratory investigations, Leflunomide-induced DRESS was considered as a final diagnosis as seen in **Figures 1** and **2**. Our patient had a RegiSCAR score of 7, which makes her a case of definite DRESS Syndrome. For the management, intravenous steroid therapy (Inj hydrocort 50 mg IV Q12H for 5 days followed by Tablet Wysolone 20 mg p/o Q12H and stopped all previous medications including antibiotics and supportive care was given for liver injury. Cholestyramine was started for the wash-out of drugs. Patient`s LFT and ferritin were improved, other symptoms also subsided including fever, the maculopapular lesions, and itching. Biopsy was advised on follow-up if any of the symptoms reappears. She was discharged on oral steroids and cholestyramine. She did not take cholestyramine and came for follow-up with worsening skin lesions but did not get readmitted, lost to follow-up. On a follow up call, the patient’s attendant informed us about death at home.

**Table 1.** Laboratory Investigations.

**CBP REPORTS**	**CASE 1**		**CASE 2**		**CASE 3**		**CASE 4**		**REFERENCE RANGE**
	**DAY 1**	**DAY 16**	**DAY 1**	**DAY 8**	**DAY 1**	**DAY 3**	**DAY 1**	**DAY 3**	
Haemoglobin	11.0	8.3	5.1	4.2	10.2	10.2	9.6	10.8	12.0–15.0gm/dl
RBC	3.7	3.1	5.1	4.2	3.9	4.1	3.5	3.9	4.5–5.5 million/mm^3^
WBC	11300	9600	38600	8400	11700	7800	15600	6900	4000–10000clls/mm^3^
Platelets	232	300	240	211	265	277	258	547	150000–410000/mm^3^
Absolute neutrophil count	7732	7296	15446	487	3744	3120	7800	4899	2000–7000 cells/mm^3^
Absolute lymphocyte count	1243	1920	14668	2772	6318	3120	5928	1518	1000–3000 cells/mm^3^
Absolute eosinophil count	2373	192	1544	0	702	624	1404	69	20–500 cells/mm^3^
Absolute monocytes count	452	0	2702	756	936	936	468	414	200–1000 cells/mm^3^
LFT									
Direct/indirect bilirubin	5.4/3.5	5.¼.0	4.1/.3.2	1.3/2.1	0.8/0.6	0.1/0.3	2.2/0.9	0.7/1.1	0–0.2mg/dl 0.2–0.8 mg/dL
SGPT/SGOT	386/583	212/162	253/154	242/67	200/94	108/50	1147/763	543/159	Up to 40 U/L/0–35 U/L
ALP	176	397	333	207	272	287	191	334	30–120U/L
Albumin	2.9	3.0	3.6	3.2	2.5	2.8	2.2	2.4	3.5–5.1g/dl

## CASE REPORT 2

**Figures 4 and 5.**
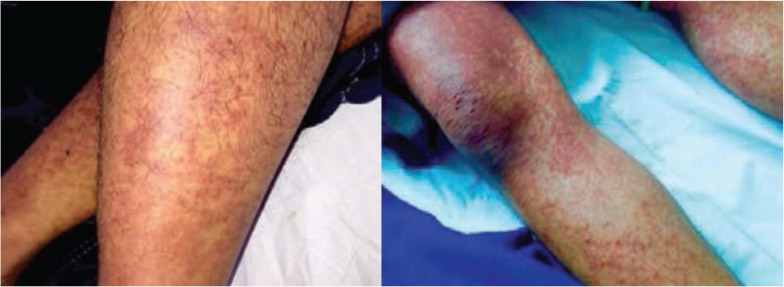
Development of rashes on lower limbs during the hospital stay.

A 39 -year-old male presented to our hospital with chief complaints of pain in abdomen, fever spikes, rashes on skin, yellowish discoloration of skin, cough. No history of DM, HTN or Hypothyroidism. Provisional diagnosis was made to be tropical infection. Scrub typhus was negative. H/O back ache, multiple joint pains for 4 years. The patient was on admax 10 mg, NSAIDS on and off (Naproxen, Sulphasalazine). On examination: Erythematous maculopapular confluent rash over the skin involving 70% BSA as shown in **Figures 4** and **5**. Eosinophilia, deranged LFT was found. Patient was treated with Inj hydrocortisone 100mg IV Q12H. Clinical and laboratory symptoms normalised after 25 days after onset of DRESS.

## CASE REPORT 3

**Figure 3.**
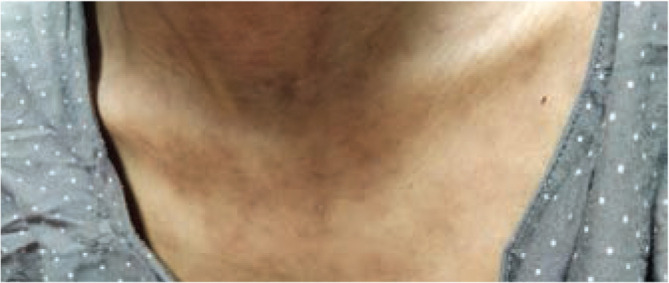
Development of rashes on the neck during the hospital stay.

A 36-year-old female presented to our hospital with fever associated with chills for 1 week, associated with joint pain, nausea, productive cough for 3 days, burning micturition for 1 month, B/L Flank pain. The provisional diagnosis was made to be acute febrile illness, UTI. On examination, the patient had rashes near the neck, pruritus (as shown in **Figure 3**), k/c/o migraine, and clinical depression. She consulted a doctor 3–4 months back with complaints of depression and social anxiety, for which Tab. Carbamazepine 200 mg p/o Q24H, Tab. Restyl 0.5 mg H/S along with other multivitamins was prescribed and was advised to continue for a month. But the patient continued for a longer time (on and off). The patient is also K/C/O RA, on Saaz 500 mg Q12H for eight months. We could attribute DRESS secondary to carbamazepine. The patient was treated with Tab. Wysolone 20 mg p/o q12h, Tab. Allegra 120mg x 4 weeks, Tab. Bilastine 20 mg p/o q24h x 3 weeks. Peripheral smear of RBC showed mild anisocytosis predominantly microcytic hypochromic leucocytosis and eosinophilia.

## CASE REPORT 4

**Figure 6.**
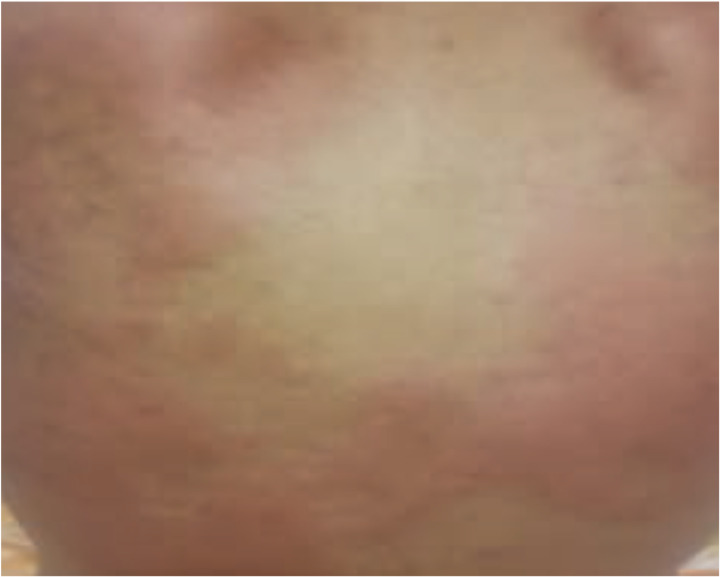
Development of rashes on back.

**Figure 7.**
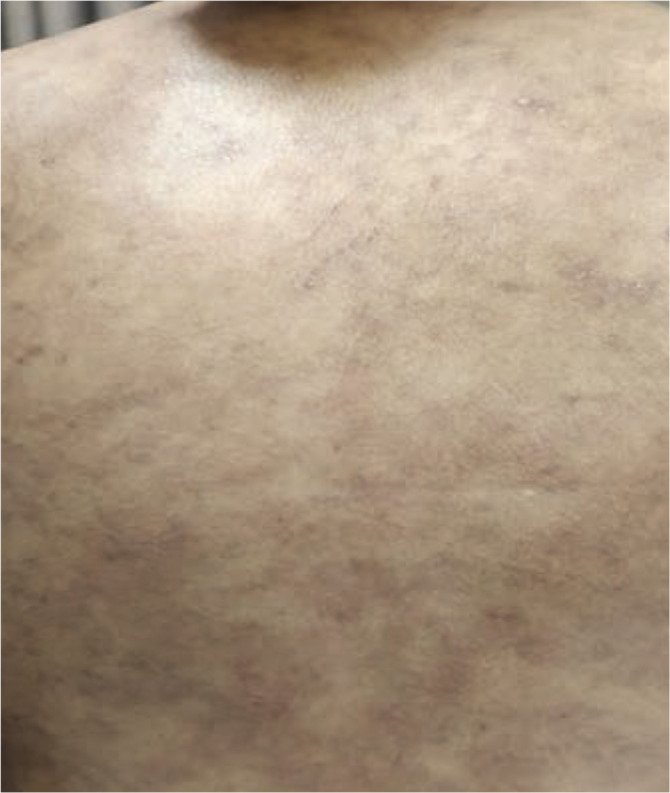
Development of rashes on back.

A 33-year-old female was admitted to our hospital with complaints of abdominal pain for 10–12 days, vomiting for 2 days, loose stools for 1 day and history of fever with chills for 17 days. She is a known case of Abdominal Lymphadenopathy first diagnosed in December 2021 (Biopsy proven: necrosis and granulomatous lymph-adenopathy, Rif sensitive) started on ATT in Jan 2022. She has a history of malar erythema, rashes all over the body (on and off) for 3–4 months (as shown in **Figures 6** and **7**), diagnosed to have ANA positive in Sep, 2021 and started on steroids since then. The rash had started from the forearm and then spread to the entire body. It was an erythematous maculopapular rash, mucosal involvement at the angles of the mouth. Lesions are associated with pain and burning sensation, lasts more than 24 hours and does not have any post inflammatory hyperpigmentation. The provisional diagnosis was made to be Neutrophilic urticaria (connective tissue disorder). The patient underwent pleurocentesis, paracentesis on day 2. Upon further investigation, the final diagnosis was made to be ATT induced DRESS with involvement of liver (Hepatitis), the patient started on IV NAC. Unfortunately, the offending drug could not be identified, as the ATT drugs were administered concurrently. Symptomatic and supportive treatment with IV and oral steroids was provided. CBP on the detailed laboratory investigations are given in **Table 1**. Clinical and laboratory symptoms normalised after 10 days of steroid therapy. The patient was discharged in a haemodynamically stable condition.

## DISCUSSION

DRESS is initiated by pyrexia with body temperature >38°C, early in the course of the disease followed by development of rashes usually maculopapular morbilliform exanthem (usually starting on the face and then generalized), with multiple follicular papules over the body mimicking pityriasis rubra pilaris.^3^ The development of rashes may vary from mild exanthem to extensive blistering and skin loss but more often a pruritic macular erythema which may contain papules, pustules, and vesicles. If the drug is not withheld, the rash may progress to erythroderma or exfoliative dermatitis. Presence of facial oedema is a characteristic presentation of DRESS; it can be the predictor of a more serious reaction.

DRESS is induced by Th2-lymphocytes and CD8+ cells. Th2 cells probably induce type IVb hypersensitivity responses affecting the skin, while CD8 + T cells cause damage to internal organs.^11^ Genetic polymorphisms of these elimination mechanisms have been implicated in several skin drug reactions, like DRESS.^12^

The Japanese Research Committee on Severe Cutaneous Adverse Reaction (J-SCAR) and the European registry on severe cutaneous adverse drug reactions (RegiSCAR) are the most commonly used clinical diagnostic criteria.^13^ RegiSCAR seems to be more accurate and comprehensive, but the J-SCAR considered viral reactivation as a diagnostic criterion in contrast to RegiSCAR. There are some other diagnostic tests for its confirmation like the lymphocyte transformation test (LTT), the intradermal test (IDT), and skin biopsy.^14^

DRESS is a severe multiorgan hypersensitivity reaction often caused by a limited number of elicited drugs in patients with genetic predisposition, genetic factors are also crucial. The risk of DRESS may be as high as 25% for individuals who have a first degree relative experiencing this syndrome. One risk factor is polymorphism in genes encoding metabolic enzymes for drugs such as CYP450 enzymes and N-Acetyltransferase. Reduction activities of metabolic enzymes causes accumulation of drugs or active metabolites which can interact with cellular proteins, evoking immune responses. Polymorphisms in HLA genes explain the genetic disposition of patients with DRESS^15^. HLA-genes are also linked to other SCAR’s other than DRESS, for example HLA-B*58:01 increase the risk for allopurinol induced DRESS and SJS/TEN. HLA-A*31:01 increase the risk for carbamazepine induced DRESS, SJS/TEN.

Viral infections could be a potential consideration at onset. In SCARs reactivation of several herpes viruses is common. It could be EBV or HHV-6 followed by HHV-7 later CMV. Reports have shown that reactivation of herpes viruses could also develop in patients with other SCARs including SJS, TEN and MPC, however reactivation of HHV-6 is found exclusively in DRESS with frequency of 43–100% (the role of HHV-6 is unique in DRESS).^15^

DRESS commonly develops two to eight weeks after starting the offending drug with a mean onset of 3 weeks. Upon rechallenge with the associated drug, symptoms may recur within one day, however symptoms may flare up 3 to 4 weeks after stopping the medicine even after initial improvement. The delayed onset of clinical symptoms of DiHS/DRESS, usually 2–6 weeks after starting therapy, often causes the potential diagnostic delay or can lead to misdiagnosis by the physician. Early withdrawal of offending drugs is needed once the diagnosis is established. The recovery from this condition has been reported to be slow, lasting for several weeks to months. The EUROPEAN RegiSCAR decided on a scoring system based on clinical features, the extent of skin involvement and the clinical course to help clinicians confirm or exclude the diagnosis of DRESS syndrome.^7^ RegiSCAR criteria is frequently used for the diagnosis of DRESS. We have categorised our cases as per RegiSCAR critera as shown in **Table 2**.

**Table 2.** RegiSCAR scoring for DRESS Syndrome.^20^

**CRITERIA**	**NO**	**YES**	**UNKNOWN**	**CASE 1**	**CASE 2**	**CASE 3**	**CASE 4**
**Fever >38°C**	−1	0	−1	**0**	**0**	**0**	**0**
**Enlarged Lymph nodes (>2 sites, >1 cms)**	0	1	0	**0**		**0**	**0**
**Atypical Lymphocytes**	0	1	0	**1**	**1**	**1**	**1**
**Eosinophilia** 700–1499 or 10–19.9%	0		0				
		1				**1**	**1**
>1500 or >20%		2		**2**	**2**		
**Skin Rash**	0		0				
Extent >50%	0	1	0	**1**	**1**	**0**	**1**
At least two: Oedema, infiltration, purpura scaling	−1	1	0	**1**	**1**	**0**	**1**
Biopsy suggesting DRESS	−1	0	0	**0**	**0**	**0**	**0**
**Internal Organ involved**	0		0				
One		1		**1**	**1**	**1**	**1**
Two or more		2					
**Resolution >15 days**	−1	0	−1	**0**	**−1**	**−1**	**−1**
**At least three biological investigations done and negative to exclude alternative diagnoses**	0	1	0	**1**	**1**	**1**	**1**
**TOTAL SCORE**				**7**	**6**	**3**	**5**

DRESS is usually accompanied with internal organ impairment. In terms of haematological changes, eosinophilia is a characteristic feature of the disease, evident in 66–95% of patients. Haematological abnormalities, especially eosinophilia and mononucleosis-like atypical lymphocytosis 27–67%, are also common. Eosinophilia can lead to infiltration of eosinophils into the tissues, which could be related to the tissue damage related to these organs. In addition, lymphadenopathy can be found in 54% of patients by physical examination. Liver injury being the most common type of organ damage found in 75–94%. Compared to other SCARs like SJS/TEN, liver injury associated with DRESS tends to be more severe and longer.^15^

### Renal involvement

Renal involvement is prevalent in 12-40% of patients with DRESS. Renal involvement in patients with DRESS is usually mild and will be recovered in time. Among the causative agents associated with renal involvement, allopurinol is found to be most common. Allopurinol induced SCAR is significantly associated with HLA-B* 58:01 allele in Han Chinese patients with gene dosage effect. Renal involvement in patients with DRESS is usually mild and will be recovered in less time. However, rarely severe interstitial nephritis, acute tubular necrosis/vasculitis may develop and lead to renal mortality.^15^

### Lung involvement

Lung involvement is the third most common type of organ involvement. It may present with impaired pulmonary function and interstitial pneumonitis, pleuritis and ARDs. Studies have shown minocycline to be associated with lung involvement; however, abacavir is associated with severe pulmonary involvement.^15^

### Cardiac involvement

Cardiac involvement is reported in 4–27% of patients with DRESS. it can be highly fatal where it occurs. They are usually present with LV dysfunction, ECG changes. medicines linked are minocycline, ampicillin, sulphonamides exhibit chest pain, dyspnoea, tachycardia, hypotension. two forms of myocarditis (self-limiting) and eosinophilic myocarditis (severe). However, neurological involvement is very rare.^15^

### Gastrointestinal and other internal organ involvements

The most frequent gastrointestinal manifestation is gastroenteritis; mucosal erosions can develop and contribute to acute bleeding. Gastrointestinal complications include chronic protein-losing enteropathy, colitis, and pancreatitis.^7^ Additional manifestations such as myositis, peripheral nerve disorders, encephalitis, meningitis, uveitis, and salivary gland inflammation may be present. Rare cases of shock and multiple organ failure have also been reported.^7^

The management involves early detection and diagnosis followed by prompt withdrawal of the offending agent. Topical corticosteroids are recommended in DRESS with no visceral organ involvement. Oral and Systemic corticosteroids are recommended in DRESS with moderate to severe visceral organ involvement.^16^ It can reduce symptoms of delayed hypersensitivity reactions.^8^ They are also known to inhibit the effect of IL-5 on eosinophil accumulation in-vivo,^7^ which may at least partly explain their benefit in the treatment of the idiopathic hypereosinophilic syndrome.^17^ Eosinophil accumulation is thought to be responsible for the internal organ involvement seen in DRESS syndrome. Immunosuppressive therapy with agents such as cyclophosphamide or cyclosporine may be even essential in cases where steroids are contraindicated or resistant. In severe DRESS, plasma exchange or intravenous immunoglobulin (IVIG) has also been used, although data on this is limited.^14^

The mainstay of treatment is the use of topical and systemic corticosteroids, but other options such as intravenous immunoglobulin, cyclosporine, mycophenolate mofetil, rituximab, and cyclophosphamide have been described.^7^

As per the Naranjo scale, the scores of each case were identified in the range of 6 to 8 and were categorised as probable adverse drug reactions (ADRs).^18^

## PREVENTION

Patch tests for antibiotics might prove effective against DRESS. An epicutaneous patch test is a useful tool for identifying the inducing agent for the DRESS syndrome.^19^ If a patch test yields a negative result and a suitable injectable form is available, then prick testing should be performed. Immediate readings should be taken at 20 minutes and delayed readings at 6 and 24 hours according to the European guidelines. If this is negative, subsequent intradermal testing is recommended.^7^ According to the Spanish guidelines for intradermal testing, the drug should be initially administered at the highest dilution (usually 1/100 of the skin prick test concentration), the interval between tests should be extended [145], different concentrations should not be tested on the same day, and special precautions should be adopted with HIV-infected patients.^7^

For primary prevention:

1. Pre-prescription Pharmacogenetic testing is recommended (HLA Screening) in identified risk populations with specific drugs to prevent DRESS.
  For example:
  - Carbamazepine: HLA*31:01 screening in Caucasian European patients and patients of Japanese origin. If positive for this allele, the use of carbamazepine may be considered if the benefits are thought to exceed the risks.
  - Phenytoin and lamotrigine: HLA-A*24:02 has been associated with DRESS syndrome induced by phenytoin or lamotrigine in the Spanish population.
  - Allopurinol: HLA B* 58:01 screening in Han Chinese, Thai, and Korean populations and descendants, especially if chronic kidney disease is present.
  - Dapsone: HLA-B*13:01 screening in patients of Asian descent.
  - Abacavir: HLA-B*57:01 screening to prevent abacavir hypersensitivity syndrome (a DRESS-like syndrome) already in routine HIV clinical practice in developed countries^7^.
2. Identification of the culprit drug is vital to prevent further cases of DRESS and the culprit drug must be notified in the allergy status of the patient and it should be brought to the patient and patient attenders’ details. Further drug allergy alerts must be recorded in the electronic medical records.^7^
3. Additionally, any case of DRESS must be notified to pharmacovigilance agencies. Promoting large collaborative networks researching projects on DRESS and including the cases in national and international registries to promote wider analysis and progression in the knowledge of DRESS and other SCARs is strongly recommended.^7^

## CONCLUSION

DRESS is a severe drug-induced hypersensitivity reaction, associated as an ADR to the drugs. Prompt identification and withdrawal of the offending agent is vital to save the patient from morbidity and mortality. Most of the patients recover completely after drug withdrawal and appropriate therapy for DRESS. However, some patients with DRESS syndrome suffer from chronic complications and die, primarily from multiple organ failure and septic shock. Controlled clinical trials investigating the most appropriate therapies and their risks are lacking, and would be invaluable in determining the optimal treatment regimen for DRESS syndrome. Furthermore, national guidelines must be developed which will help the clinicians to diagnose DRESS and use the most appropriate therapy.
